# Bilateral co-secretory lesions presenting with coexisting Cushing syndrome and primary aldosteronism: a case report

**DOI:** 10.1186/s12902-023-01454-8

**Published:** 2023-11-29

**Authors:** Hongjiao Gao, Li Li, Fei Chen, Yan Ren, Tao Chen, Haoming Tian

**Affiliations:** 1 Department of Endocrinology and Metabolism, Adrenal Center, West China Hospital of Sichuan University, Chengdu, 610041 Sichuan China; 2 Department of Endocrinology and Metabolism, The Third Affiliated Hospital of Zunyi Medical University (The First People’s Hospital of Zunyi), Zunyi, Guizhou China; 3 Institute of Clinical Pathology, West China Hospital of Sichuan University, Chengdu, Sichuan China

**Keywords:** Cushing’s syndrome, Primary aldosteronism, Adrenal vein sampling, Immunohistochemistry, Aldosterone-producing cell cluster

## Abstract

**Background:**

There is an increasing number of cases of aldosterone- and cortisol-producing adenomas (A/CPAs) reported in the context of primary aldosteronism (PA). Most of these patients have PA complicated with subclinical Cushing's syndrome; cases of apparent Cushing's syndrome (CS) complicated with aldosteronism are less reported. However, Co-secretory tumors were present in the right adrenal gland, a cortisol-secreting adenoma and an aldosterone-producing nodule (APN) were present in the left adrenal gland, and aldosterone-producing micronodules (APMs) were present in both adrenal glands, which has not been reported. Here, we report such a case, offering profound insight into the diversity of clinical and pathological features of this disease.

**Case presentation:**

The case was a 45-year-old female from the adrenal disease diagnosis and treatment centre in West China Hospital of Sichuan University. The patient presented with hypertension, moon-shaped face, central obesity, fat accumulation on the back of the neck, disappearance of cortisol circadian rhythm, ACTH < 5 ng/L, failed elevated cortisol inhibition by dexamethasone, orthostatic aldosterone/renin activity > 30 (ng/dL)/(ng/mL/h), and plasma aldosterone concentration > 10 ng/dL after saline infusion testing. Based on the above, she was diagnosed with non-ACTH-dependent CS complicated with PA. Adrenal vein sampling showed no lateralization for cortisol and aldosterone secretion in the bilateral adrenal glands. The left adrenocortical adenoma was removed by robot-assisted laparoscopic resection. However, hypertension, fatigue and weight gain were not alleviated after surgery; additionally, purple striae appeared in the lower abdomen, groin area and inner thigh, accompanied by systemic joint pain. One month later, the right adrenocortical adenoma was also removed. CYP11B1 were expressed in the bilateral adrenocortical adenomas, and CYP11B2 was also expressed in the right adrenocortical adenomas. APN existed in the left adrenal gland and APMs in the adrenal cortex adjacent to bilateral adrenocortical adenomas. After another surgery, her serum cortisol and plasma aldosterone returned to normal ranges, except for slightly higher ACTH.

**Conclusions:**

This case suggests that it is necessary to assess the presence of PA, even in CS with apparent symptoms. As patients with CS and PA may have more complicated adrenal lesions, more data are required for diagnosis.

## Background

Because both adrenal Cushing's syndrome and primary aldosteronism (PA) can manifest as adrenocortical adenomas, it is difficult to distinguish between them on the sole basis of adrenal computed tomography (CT). There may also be multiple adenomas with different functions in the same adrenal gland [1], which also leads to the difficulty in the interpretation of adrenal vein blood collection results. With the increased reports on cases of PA complicated with subclinical Cushing's syndrome in clinical practice, increasing attention is being given to the screening of PA complicated with subclinical Cushing's syndrome. However, PA screening may be ignored in the diagnosis and treatment of adrenal Cushing's syndrome.

Although it has been reported that PA with a diameter > 2 cm may be complicated with aldosterone- and cortisol-producing adenomas (A/CPAs) [2], cases of apparent Cushing's syndrome complicated with PA are less well known.

Recently, Y. Fushimi et al. [3] reported a case of apparent Cushing's syndrome complicated with PA. The cortisol-producing enzyme cytochrome P450 (CYP) 11B1 was diffusely expressed in the adenoma, but based on staining, the aldosterone synthase CYP11B2 was significantly expressed in the adjacent adrenal cortex. This finding indicated that aldosterone-producing micronodules (APMs) in the adjacent adrenal cortex may be the pathological basis of PA.

Here, a case of bilateral co-secretory lesions presenting with coexisting Cushing syndrome and primary aldosteronism detected by AVS and confirmed by immunohistochemical analysis after surgical resection is reported. Moreover, APMs were found in the adrenal cortex adjacent to bilateral adrenocortical adenomas; an aldosterone-producing nodule was detected adjacent to the unilateral adenoma.

## Case presentation

**Fig. 1:**
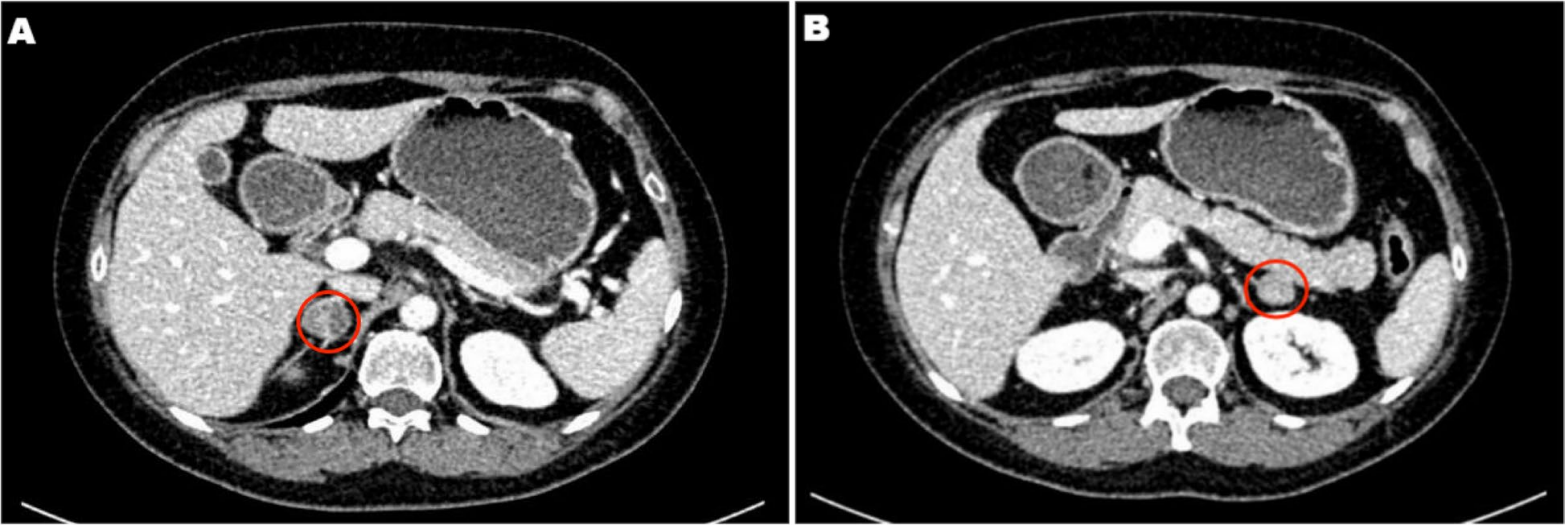
Adrenal CT of the patient: A nodule with a size of approximately 1.6 × 1.5 cm was found in the left adrenal gland, and a nodule with a size of approximately 2.2 × 1.8 cm was found in the right adrenal gland. Irregular mild to moderate enhancement was on enhanced CT, and the surrounding fat gap was clear

A 45-year-old female patient was admitted to the adrenal disease diagnosis and treatment centre in West China Hospital of Sichuan University due to "increased blood pressure, weight gain for one year and facial oedema for half a year". After nifedipine controlled-release tablets 30 mg daily and terazosin 2 mg daily were applied, the blood pressure of this patient was still as high as 179/113 mmHg. She had no family history of endocrine disease or malignant tumour. Her body mass index (BMI) was 25.6 kg/m^2^ at admission, with a moon-shaped face, fat accumulation on the back of the neck and thin skin. Hormonal, glucose, renal function, lipid, and blood electrolyte tests were completed, and the physiological rhythm of cortisol had disappeared. Aldosterone-renin-angiotensin system (RAAS) results showed a significant decrease in renin activity and a significantly higher aldosterone/renin ratio (ARR) (as provided in Table 1). Dynamic testing for hormones was conducted, and the results were as follows: (i) in terms of the saline infusion test (SIT) in supine position, the before and after aldosterone level was 17.03 ng/dL and 15.45 ng/dL, respectively; (ii) in terms of the captopril challenge test (CCT), the before and after aldosterone level was 18.49 ng/dl and 15.25 ng/mL, respectively, with an inhibition rate of 17.52%; (iii) in terms of the standard low-dose dexamethasone suppression test, the before and after serum cortisol level was 467.9 nmol/L and 786.3 nmol/L, respectively; the before and after 24-h urine free cortisol (24-h UFC) level was 332.3 µg/24 and 480.4 µg/24, respectively. An enhanced CT scan revealed adenoma lesions in both adrenal glands (Fig. 1a and b). Bone mineral density measurement with dual-energy X-ray absorptiometry indicated osteoporosis. Chest CT showed old fractures of the 9th rib on the left side and the 2nd rib on the right side.

**Table 1:** Peripheral blood laboratory data for this case

Metabolism and Electrolytes(reference range)		Endocrine markers(reference range)	
BG	4.45(3.9–5.9)mmol/L	serum cortisol (08:00)	467.9 (147.3–609.3) nmol/L
Triglycerides	1.93(0.29–1.83)mmol/L	serum cortisol (16:00)	498.5 nmol/L(64–340)nmol/L
Cholesterol	8.18(2.8–5.7)mmol/L	serum cortisol (00:00)	411.9(< 50)nmol/L
HDL-C	1.89(> 0.9)mmol/L	24 h UFC	332.3(20.3–127.6)ug/24 h
LDL-C	5.69(< 4.0)mmol/L	ACTH	1.96(5.0–78)ng/L
UREA	3.19(2.95–7.7)mmol/L	PAC	16.79(9.8–27.5)ng/dl
Creatinine	57(48–79)µmol/L	PRA	0.05 (0.93–6.56) ng/mL/h
Potassium	3.66(3.5–5.3)mmol/L	ARR	335.8(ng/dl)/(ng/mL/h)
Sodium	141.0(137–147)mmol/L	Epinephrine	64(60–104)ng/L
Calcium	2.09(2.11–2.52)mmol/L	Noradrenaline	138(174–357)ng/L
Phosphorous	0.99(0.81–1.45)mmol/L	PTH	7.37(1.60–6.90)pmol/L
Magnesium	1.0(0.67–1.64)mmol/L	25-OH-VD	19.6(47.7–144)nmol/L

*Abbreviations*: *BG* blood glucose, *LDL-C* high-density lipoprotein cholesterol, *LDL-C* low-density lipoprotein cholesterol, *24 h UFC* 24-h urine free cortisol, *ACTH* adrenocorticotropic hormone, *PAC* plasma aldosterone concentration, *PRA* plasma renin activity; ARR aldosterone/renin ratio

Based on the above clinical features, the patient was diagnosed with "non-ACTH-dependent Cushing's syndrome complicated with PA". To assess lateralization, adrenal vein sampling (AVS) stimulated by ACTH was performed after obtaining informed consent. The results showed no lateralization of cortisol and aldosterone secretion (Table 2).

**Table 2:** Results of AVS

	Vena cava	Left adrenal vein -1	Right adrenal vein -1	Left adrenal vein -2
ALD (ng/dL)	15.97	162.1	162.37	183.18
serum cortisol (nmol/L)	1209	49735	53921	45548
ALD/PTC	0.0132	0.0033	0.0030	0.0040

**Fig. 2:**
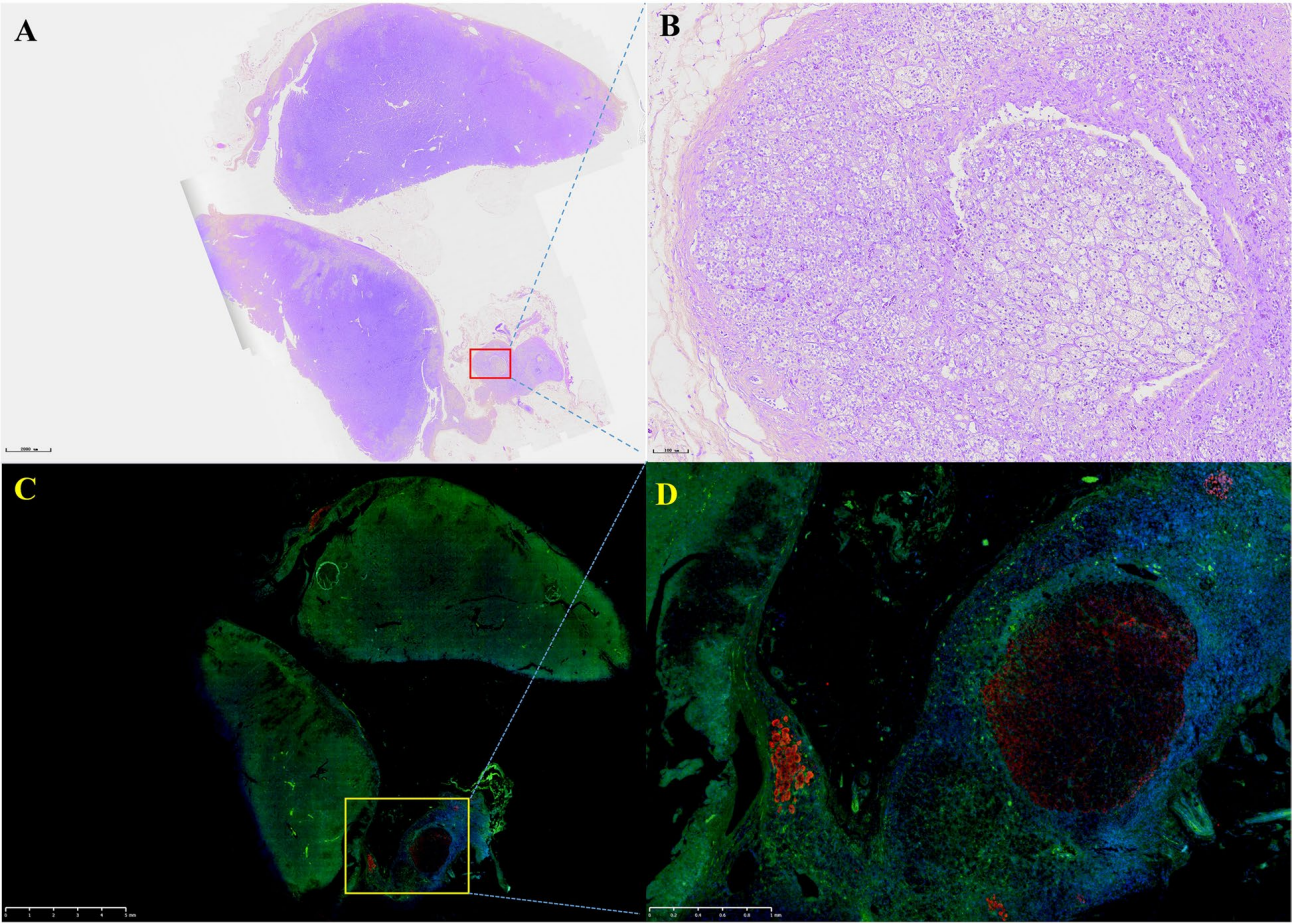
Routine hematoxylin and eosin (H&E) staining and immunofluorescence of the left adrenocortical adenoma (green represents expression of CYP11B1 and red that of CYP11B2). This adrenocortical adenoma and the surrounding cortex was cut into three parts. **A** and **C** show the overall appearance of the resected portion, with a nodule adjacent to the adenoma. **B** shows a neoplastic lesion formed by clear cells (aldosterone-producing cell) within nodules, lacking a fibrous envelope. **C** clearly shows the weak and diffuse expression of CYP11B1 in adrenocortical adenoma and CYP11B2 expression in a nodule in the cortex adjacent to the adenoma. **D** shows local enlargement of the aldosterone-producing nodule and three aldosterone-producing micronodules adjacent to it

**Fig. 3:**
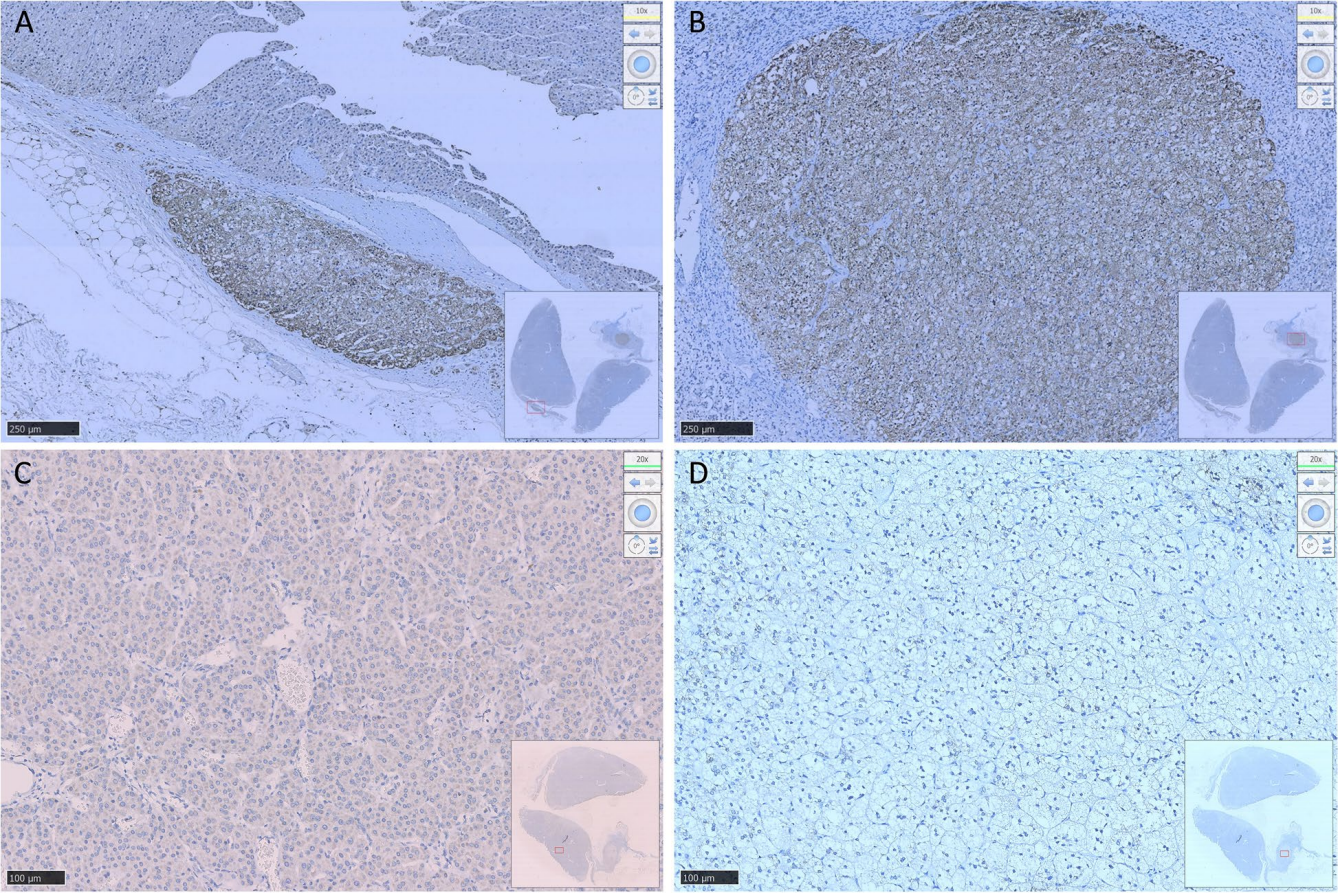
Resected adrenocortical adenoma and part of the adrenal cortex on the left side. **A** shows expression of Aldosterone-producing micronodule CYP11B2 in the cortex adjacent to the adenoma. **B** shows an aldosterone-producing nodule with a diameter of approximately 2 mm. **C** shows weak positive expression of CYP11B1 in the adenoma and **D** negative expression of CYP11B1 in the aldosterone-producing nodule

**Fig. 4:**
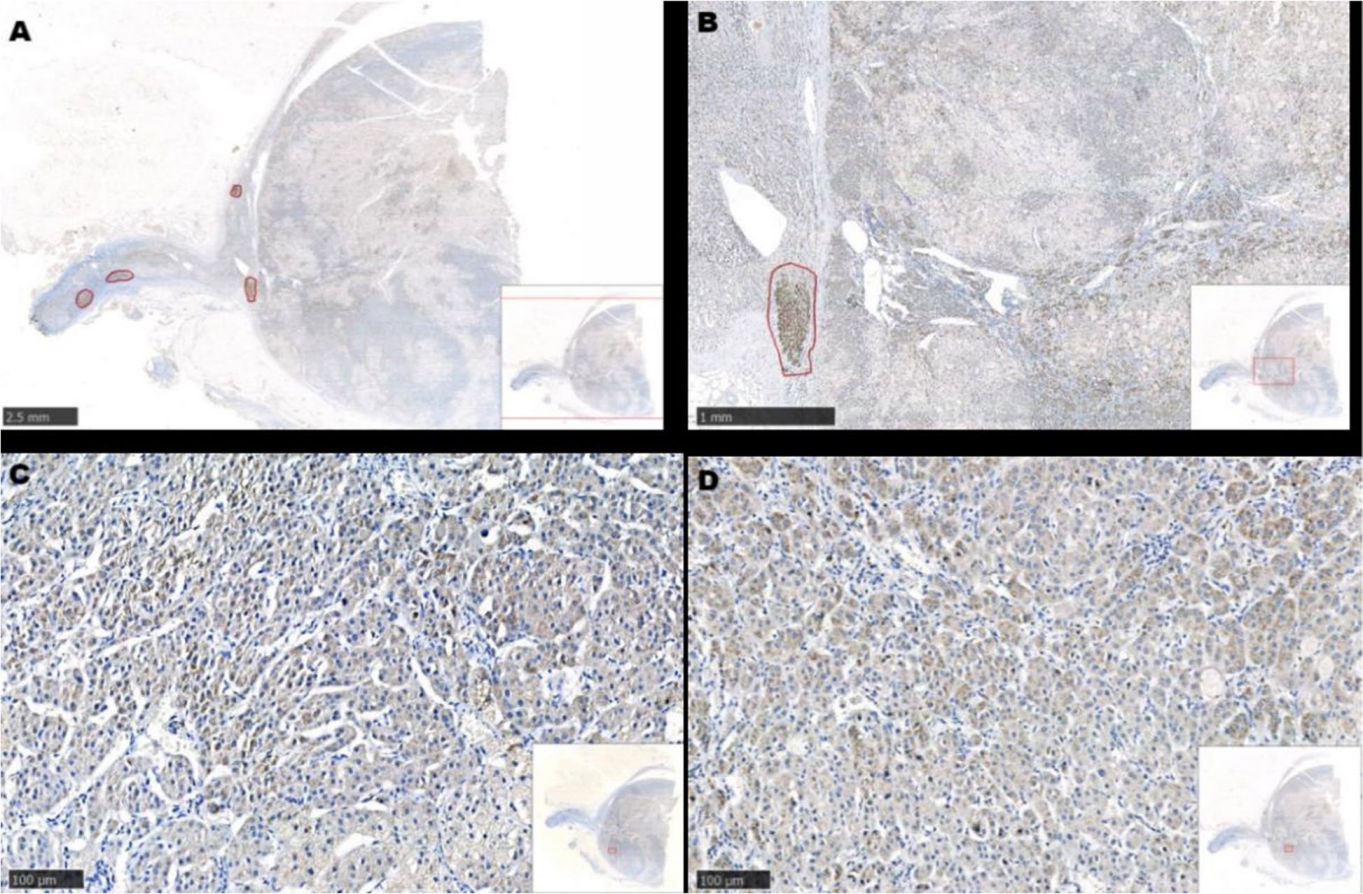
Resected adrenocortical adenoma and part of the adrenal cortex on the right side. **A** and **B** show several Aldosterone-producing micronodules (positive expression of CYP11B2) in the cortex adjacent to the adenoma. **C** shows diffuse expression of CYP11B1 in the adenoma. **D** shows diffuse expression of CYP11B2 in the adenoma

After communicating with the patient, the left adrenocortical adenoma was first removed by robot-assisted laparoscopic resection; the thickened adrenal cortex near the left adrenocortical adenoma was also resected during the surgery. The pathological report revealed adrenocortical adenoma, the Weiss score was 1, and immunohistochemistry showed weak CYP11B1 expression in the adenoma and positive CYP11B2 expression in an adjacent nodule. Hypertension was not alleviated after surgery. One month later, purple lines appeared on both sides of the lower abdomen, groin area and inner thigh, accompanied by weight gain, apparent systemic joint pain and fatigue in both lower limbs. The patient was readmitted to the hospital, and examination revealed orthostatic ALD at 11.99 ng/dL, PRA at 0.08 ng/mL/h, angiotensin II at 39.38 ng/L (reference range: 55.3–115.3 ng/L) and ARR at 149.88 (ng/dL)/(ng/mL/h). In addition, ACTH was 2.37 ng/L, serum cortisol was 352.30–353.50–283.90 nmol/L at 8 h-16 h-24 h, 24-h UFC was 112.8 µg, and serum cortisol was 342.10 nmol/L in the morning after the 1 mg dexamethasone suppression test. Enhanced CT of the kidneys and adrenal glands showed no solid nodules or masses in the left adrenal gland, though a nodule with a size of approximately 2.2*1.8 cm was detected in the right adrenal gland. Enhanced CT showed irregular mild to moderate enhancement. Therefore, the diagnosis was still "non-ACTH-dependent Cushing's syndrome complicated with PA". Subsequently, the right adrenocortical adenoma and the thickened adrenal cortex near the right adrenocortical adenoma were removed by robot-assisted laparoscopic resection. The pathological report indicated adrenocortical adenoma, and immunohistochemistry showed diffuse homogeneous expression of CYP11B1 and CYP11B2. Antibodies against CYP11B1 (MABS502) and CYP11B1 (MABS1251) were purchased from the Millipore Corporation. There were APMs in the adrenal cortex adjacent to the bilateral cortical adenomas. The fluorescence staining image of the left cortical adenoma is shown in Fig. 2. The immunohistochemistry image of the left adrenal gland is given in Fig. 3 and that of the right adrenal gland in Fig. 4. The immunofluorescence method used in this study was indirect immunofluorescence double staining procedure. Paraffin-embedded human adrenal tissues were prepared using heat-induced epitope retrieval after deparaffinization. Tissue sections were blocked with 5% goat serum in PBS, pH 7.4, containing 0.5% SDS, for 1 h. The slides were incubated with individual primary antibodies at 4℃ overnight, followed by incubation with Alexa Fluor 488-, and Alexa Fluor 647-conjugated secondary antibodies specific to the species of the primary antibodies with DAPI for immunofluorescence staining. Antibodies used included anti-CYP11B1 (Millipore, Cat. No. MABS502, 1:100), anti-CYP11B2(Millipore, Cat. No. MABS1251, 1:100), Alexa Fluor 488-conjugated anti-rat IgG secondary antibody (CYP11B1; Green) and Alexa Fluor 647-conjugated anti-mouse IgG secondary antibody (CYP11B2; Red). Nuclei were stained with DAPI.

The Cushing's syndrome in this patient disappeared after surgery, and glucocorticoids were discontinued after 15 months according to medical advice. Follow-up was conducted for half a year after drug discontinuance, and the patient had no fatigue or dizziness; she was satisfied with the outcomes. Her systolic and diastolic blood pressure remained at 100–120 mmHg and 70–80 mmHg, respectively. During the most recent re-examination, the following results were obtained: (1) orthostatic ALD of 19.1 ng/dL and orthostatic renin concentration of 12.59 µIU/mL, with an aldosterone/renin ratio (ARR) of 1.52; (2) PTC at 8 AM of 247 nmol/L, ACTH of 93.55 ng/L and 24-h UFC of 26.8 µg; (3) parathyroid hormone of 3.86 pmol/L; (4) 25-OH-VitD of 119.5 nmol/L; (5) serum creatinine of 60 µmol/L; (6) serum sodium of 140.4 nmol/L, serum potassium of 3.87 mmol/L and serum calcium of 2.27 mmol/L.

## Discussion and conclusions

Adrenal Cushing's syndrome is caused by excessive autonomic secretion of cortisol induced by adrenal cortical tumours or adrenal cortical hyperplasia; primary aldosteronism (PA) is caused by excessive autonomic secretion of aldosterone induced by adrenal cortical tumours or adrenal cortical hyperplasia. More adverse symptoms occur if aldosterone and cortisol-producing adenomas are present. Specifically, (1) it is more difficult to control hypertension; (2) the incidence of major adverse cardiovascular and cerebrovascular events would increase [4]; (3) glucose intolerance and other metabolic complications would be aggravated [5, 6]; (4) patients would be prone towards osteoporosis [7, 8]; (5) adrenal vein sampling results may be misinterpreted [9]; and (6) adrenal insufficiency may occur after surgery. Therefore, it is of great clinical significance to avoid missed diagnosis of A/CPAs.

Despite many reports on A/CPAs, the majority of these patients may have subclinical Cushing's syndrome (SCS), and cases of apparent Cushing's syndrome complicated with PA are rarely reported. In the present case, the clinical manifestation of Cushing's syndrome were more apparent, and it would be appropriate to call it cortisol-aldosterone cosecretoma. Naoyoshi Onoda et al. [10] reported a case of Cushing's syndrome caused by a left adrenocortical adenoma (30 mm in diameter) and PA caused by a right adrenocortical adenoma (20 mm in diameter), and Fushimi et al. [3] reported a case of right A/CPA (25 mm*22 mm in size). Interestingly, in the present report, the patient had bilateral A/CPAs, and the clinical manifestations of Cushing's syndrome became more apparent after unilateral resection was performed. Similar to the above two cases, APMs were found in the adrenal cortex adjacent to the A/CPAs, but aldosterone-producing nodules were found near the cortisol-producing adenoma on the left side.

The biochemical phenotype of APM-inducing autonomic aldosterone secretion has not been clarified. APMs can also be found in the adrenal tissue of 30% of individuals with normal blood pressure [11] and surrounding areas of APA [12, 13]. APMs do not express CYP11B1 or CYP17A1, which are necessary for the generation of cortisol [12, 14]. In our patient, the aldosterone-producing nodule in the left adrenal gland may have developed from APM. More than one-third of APMs carry known mutations in CACNA1D and ATP1A1, promoting the generation of aldosterone [14, 15]. Unfortunately, we did not perform whole-exome sequencing on the DNA of the peripheral blood and adenoma tissues of this patient. Due to the existence of APMs adjacent to the adenoma, it remains unclear whether there is a risk of the relapse of PA in these cases after resection of adrenal the adenoma. Therefore, it was necessary to conduct medical follow-up for this patient.

Remi Goupil et al. performed AVS on 8 patients with cortisol-producing adenoma (CPA), and the results showed that cortisol on the CPA side was higher than that on the contralateral side (median, 6.7 times [range: 2.4–27.2]); *P* = 0.012]) [16]. There was no significant difference in bilateral cortisol and aldosterone concentrations after AVS in this patient, which is consistent with bilateral A/CPA. Although immunohistochemical results revealed weak expression of CYP11B1 for the first time, expression of cortisol in bilateral adrenal venous blood samples increased significantly after ACTH stimulation. Hence, cortisol was over-synthesized on both sides, and bilateral A/CPAs was definitively diagnosed.

In summary, this case highlights the need for A/CPA screening. The complicated pathological features of these cases impose challenges to our understanding of this disease. Due to the presence of APMs in the adrenal cortex near bilateral adrenocortical adenomas, more clinical data are required to identify whether the disease might relapse after simple resection of the adenoma in these patients. Therefore, further medical follow-up of these patient is needed.

## Data Availability

Not applicable.

## Abbreviations

CS: Cushing's syndrome
PA: Primary aldosteronism
ACTH: Adrenocorticotropic hormone
UFC: Urinary free cortisol
AVS: Adrenal vein sampling
A/CPA: Aldosterone-and cortisol producing adenoma
APN: Aldosterone-producing nodules
APM: Aldosterone-producing micronodule
CYP: Cytochrome P450
CT: Computed tomography
PAC: Plasma aldosterone concentration
PRA: Plasma renin activity
ARR: Aldosterone /renin ratio
